# Oyster aquaculture impacts *Zostera marina* epibiont community composition in Akkeshi-ko estuary, Japan

**DOI:** 10.1371/journal.pone.0197753

**Published:** 2018-05-24

**Authors:** Carter S. Smith, Minako Ito, Mizuho Namba, Masahiro Nakaoka

**Affiliations:** 1 Institute of Marine Sciences, University of North Carolina at Chapel Hill, Morehead City, United States of America; 2 Graduate School of Environmental Science, Hokkaido University, Akkeshi, Hokkaido, Japan; 3 Akkeshi Marine Station, Field Science Center for Northern Biosphere, Hokkaido University, Akkeshi, Hokkaido, Japan; Bigelow Laboratory for Ocean Sciences, UNITED STATES

## Abstract

Coastal fisheries are in decline worldwide, and aquaculture has become an increasingly popular way to meet seafood demand. While finfish aquaculture can have substantial adverse effects on coastal ecosystems due mostly to necessary feed inputs, bivalves graze on natural phytoplankton and are often considered for their positive ecosystem services. We conducted two independent studies to investigate the effects of long-line *Crassostrea gigas* oyster aquaculture on *Zostera marina* seagrass beds and associated epibiont communities in Akkeshi-ko estuary, Japan. Results from both studies yielded no evidence of an effect of oyster aquaculture on the morphology, density, or biomass of *Z*. *marina*, but significant differences were apparent in the epibiont community. Reference seagrass beds located away from aquaculture had higher seagrass epiphyte loads and higher abundances of amphipods. Conversely, seagrass beds below aquaculture lines had higher sessile polychaete biomass and higher isopod abundances. Our results suggest that the presence of oyster aquaculture may have indirect effects on seagrass by changing epibiont community composition and relative abundances of species. One proposed mechanism is that cultured oysters feed on epiphytic diatoms and epiphyte propagules before they can settle on the seagrass, which reduces epiphyte loads and influences subsequent faunal settlement. If carefully implemented and monitored, long-line oyster aquaculture may be a sustainable option to consider as bivalve aquaculture expands to meet global seafood demand, but further work is needed to fully assess and generalize the community-level effects on seagrass epibionts.

## Introduction

Marine fisheries are in decline [1, 2] and over the last several decades aquaculture has become an increasingly viable option for meeting growing seafood demands [3]. Globally, aquaculture is the fastest expanding food-producing sector and currently generates over 50% of the world’s seafood for human consumption [4]. This raises the question of how to sustainably expand aquaculture enterprises without adding additional stressors to already degraded coastal systems [5].

The adverse effects of aquaculture on coastal ecosystems have been well studied and documented and they include habitat loss [6–8], wild fish stock compromises [9, 6], sediment enrichment [10], and bottom disturbance [11, 12]. Many of these negative effects, however, are most prominently associated with finfish and shrimp aquaculture. Of the nearly 600 marine species that are cultured globally, bivalves make up roughly 20% of production by weight [4] and have a high potential for sustainability because: 1) bivalves have a low trophic position, which increases production efficiency [6, 13]; and, 2) most bivalves filter-feed directly from the water column, which limits the environmental impact near the farm and also alleviates pressure on wild stocks that are sometimes used as feed [14]. There is a common misperception that all aquaculture is equally bad [14], yet cultured bivalves may provide a variety of positive services to coastal habitats, such as reducing eutrophication and enhancing water quality [15]. It is precisely because of these positive services that bivalve restoration has become an important priority in many areas of the world [16].

Of the multitude of bivalve species cultured worldwide, *C*. *gigas* (Pacific oyster) is the most dominant, with a global value of nearly 4 billion USD per year [17]. Like other forms of aquaculture, however, oyster culture has inherent conflicts with a variety of other economic, social, and ecological interests [18]. The impacts of oyster aquaculture on seagrass beds are of particular concern because the sites that are typically chosen for Pacific oyster culture (e.g., relatively shallow coastal embayments) often overlap seagrass distribution. Seagrass is recognized as a critical nursery habitat for commercially and recreationally important fish [19], and it also provides many other services, such as sediment stabilization, wave amelioration, and carbon sequestration [20]. Furthermore, the global extent of seagrass beds has declined precipitously in recent decades [21].

Peterson & Heck [22] showed that the presence of tulip mussels reduced seagrass epiphyte loads resulting in 10% less light limitation. Similarly, Wall *et al*. [23] found that *Z*. *marina* leaf productivity was highest in experimental treatments with the densest bivalves versus controls with no bivalves. Accordingly, Peterson *et al*. [24] proposed that bivalve aquaculture could actually promote the expansion of seagrass by filtering the water, thereby reducing water column turbidity in locations where light is limiting. The effects of bivalves on seagrass can be complex and both system and organism dependent [25, 23], but looking to the future, it will be important to capitalize on these kinds of facilitative interactions as coastal aquaculture enterprises inevitably expand; in order to do this, a better understanding of the direct and indirect effects of aquaculture will be needed.

The vast majority of oyster aquaculture impact studies on seagrass have been conducted in North American waters [25–30], despite the fact that the bulk of aquaculture production comes from Asia (where *C*. *gigas* is native). Japan, in particular, has experienced declines in catches of major coastal fishery species and it is now one of only three countries characterized as a net importer of seafood [31, 32]. Nearly 80% of the Japanese population is coastal [33], which has dictated much of Japan’s cultural identity and led to a historic reliance on marine production as the major protein source for the population; the resulting per capita consumption of fish in Japan is 50.2 kg yr^-1^, which is two and a half times the world average [4].

In this paper, we present the results of two field surveys assessing the impacts of long-line *C*. *gigas* oyster aquaculture on *Z*. *marina* morphology and its associated epibiont community in Akkeshi-ko estuary, Japan. Specifically, we address the following questions: 1) does long-line oyster aquaculture affect *Z*. *marina* morphology, density, and biomass; and, 2) does the presence of oyster aquaculture have an effect on the epiphyte and epifaunal communities living on the seagrass. By pursuing these questions, we looked beyond exclusively assessing the direct effects of aquaculture on seagrass, and also included an investigation into changes in the epibiont community that might have indirect impacts on the seagrass. In principle, aquaculture may be a sustainable means for producing a vital protein source without depleting wild fishery stocks; however, in practice, more research is needed to identify and quantify the impacts of aquaculture on coastal ecosystems and to determine best management practices.

## Methods

### Study area and survey design

**Fig 1:**
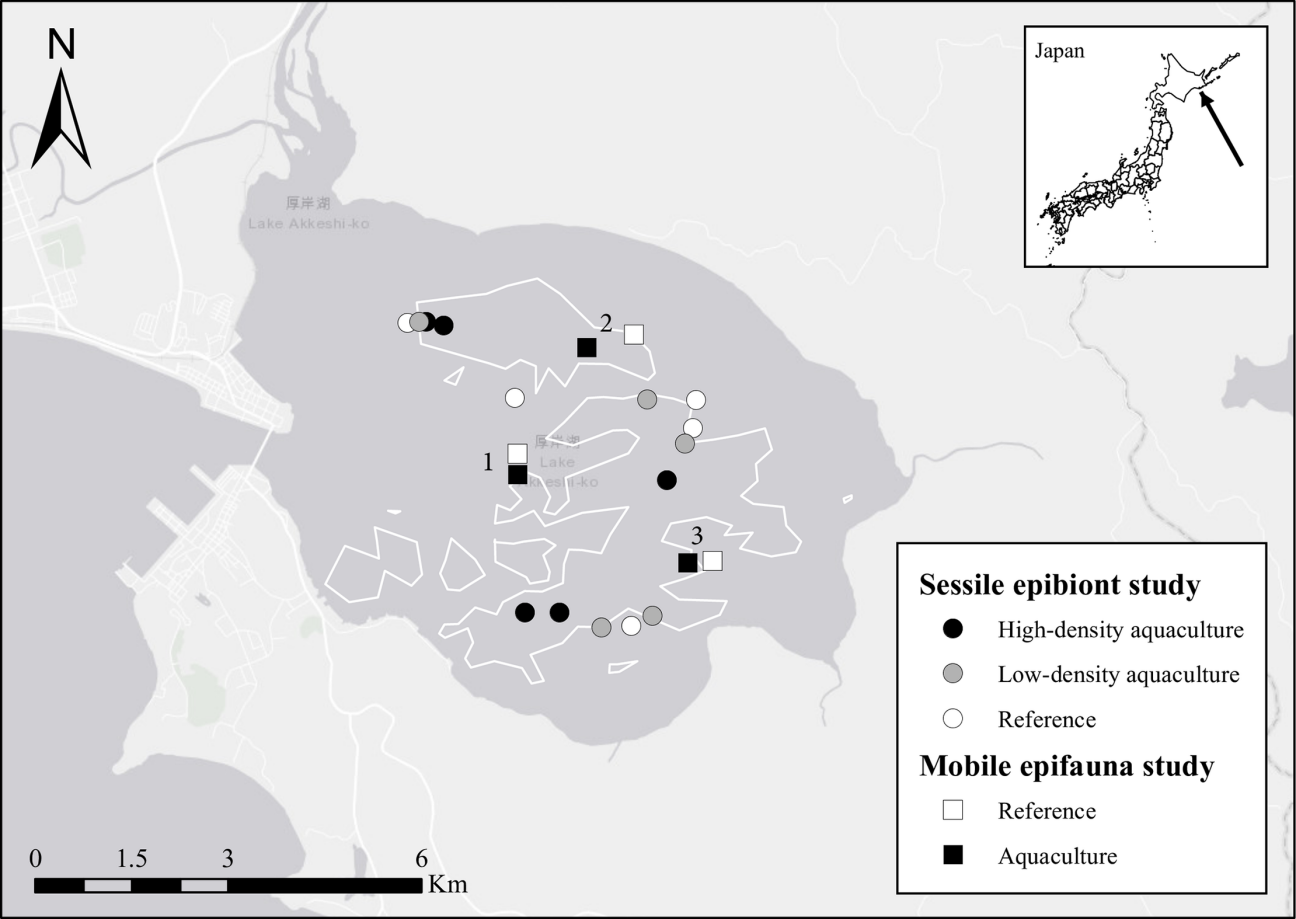
Site locations in Akkeshi-Ko estuary, Hokkaido, Japan. Aquaculture areas are roughly outlined in white, based on 2011 Esri aerial imagery. Numbers indicate regions for the mobile epifauna study.

**Fig 2:**
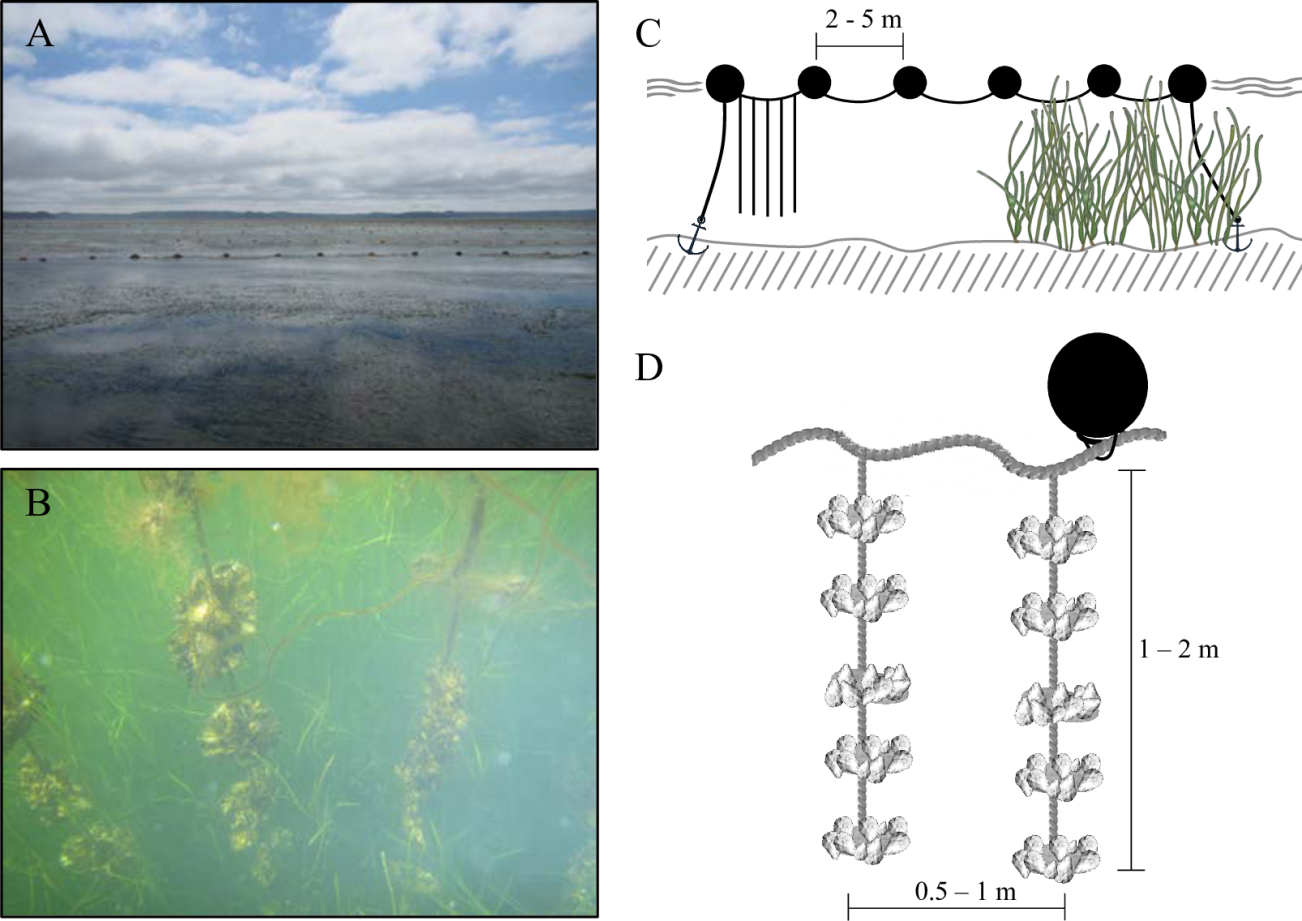
Photographs and schematics of long-line oyster aquaculture. Photograph of the surface of Akkeshi-ko estuary (A) and underwater photograph of long-line aquaculture in Hokkaido, Japan (B). Schematic of long-line oyster aquaculture (C) and close-up of individual aquaculture lines (D).

Our study was conducted in Akkeshi-ko estuary, which is one of the largest estuarine systems in Hokkaido, Japan (surface area, SA = ~32 km^2^; 43°01–43°04N, 144°50–144°56E; Fig 1). Akkeshi-ko estuary is connected to Akkeshi Bay by a narrow channel and receives freshwater inflow from two rivers. The estuary is shallow, with an average depth of less than 2 m, and water flow is driven by wind and tide. The majority of Akkeshi-ko is covered with two species of seagrass (*Z*. *marina* in the subtidal and *Zostera japonica* in the intertidal) [34], but oyster aquaculture is exclusively conducted over subtidal areas. It is important to note that *Z*. *marina* in Akkeshi-ko is unusually large with canopy heights that are consistently over 3 m and reach all the way to the surface even at high tide (Fig 2A); therefore, the aquaculture gear regularly comes into contact with the seagrass (Fig 2B).

Natural oysters have been harvested in Akkeshi-ko for over 200 years, but in recent decades there has been an expansion of oyster and clam cultivation and roughly one half of the estuary is currently allocated to aquaculture (Fig 1). There are several techniques in use, but the predominant method is long-line oyster culture, whereby lines of rope are suspended from buoys along parallel surface transects (Fig 2A and 2C), with neighboring transects typically separated by 15 m or more. Shorter (1–2 m) lines dangle down between the buoys with clumps of oysters (spaced approximately every 30 cm vertically) grown on scallop shells attached along the lines, but these lines never come into contact with the bottom (Fig 2B and 2D)[35]. Oyster production varies across the estuary, based on the stocking technique used by individual fishers; on average, 500 mature oysters are produced per vertical line, which is equivalent to roughly 500 oysters m^-2^ (personal observation M. Ito). This stocking density is consistent with typical commercial harvest areas [36]. Oyster yield for all of Akkeshi-ko is roughly 200 tons yr^-1^ [37].

To determine impacts of *C*. *gigas* aquaculture on seagrass, we conducted two independent studies in summer 2016. First, we surveyed seagrass beds in Akkeshi-ko in July using a stratified random sampling design. We subsequently conducted a mobile epifauna study in August to further characterize differences in the epifaunal community associated with seagrass inside and outside of culture operations and also to control for some of the variation we saw among sites in the first study. The Hokkaido prefecture government regulates the collection permission for aquatic organisms in Akkeshi-ko estuary. According to the regulations, the collection of eelgrass and non-commercial invertebrate species by the methods we used (i.e., hand-towed mesh bags) was exempted from needing a permit. Furthermore, sampled plants and invertebrates did not include any endangered or protected species.

### Sessile epibiont study

In July 2016, we sampled 15 sites in Akkeshi-ko that were stratified between, but randomly sampled within, aquaculture and non-aquaculture areas (Fig 1). All sites were between 0.9 m and 1.6 m deep. Five of the sites were reference seagrass beds that were at least 100 m from any aquaculture buoys based upon reference distances specified in several other bivalve aquaculture impact studies [38, 39], as well as results from Skinner *et al*. [29] that hanging-bag oyster aquaculture effects on seagrass were undetectable at 100 m. Before conducting any analyses, we categorized the remaining 10 aquaculture sites as either low-density (i.e., edge aquaculture) or high-density (i.e., interior aquaculture); low-density sites were individual isolated lines or lines at the edge of aquaculture patches, whereas high-density sites had at least four lines in the surrounding 100 m of water. At each of the 15 sites (5 of each treatment for a balanced design) we collected: 1) 5 density and morphology samples; and, 2) 5 sessile epibiont samples. For the density and morphology samples, we collected all seagrass shoots present inside of five randomly placed 50 x 50 cm quadrats at each site. For the sessile epibiont samples, we randomly selected five individual seagrass blades (independent of the density and morphology samples) that were floated into Ziploc bags to ensure minimal loss of sessile organisms. All aquaculture samples were taken beneath aquaculture lines (given that lines were free hanging and some movement was possible, we defined this as within one meter of culturing lines). Samples were stored in dark covered containers on the boat and transported to the refrigerator at the Akkeshi Marine Station within six hours of collection.

To characterize density we enumerated shoots per quadrat and for morphology we measured leaf height and blade width from the first five shoots in each quadrat (*sensu* [40]). Above-ground biomass was obtained by manually removing all epibionts from grass blades and drying at 60°C to constant weight. To process the sessile epibiont samples, each individual seagrass shoot was scraped of epibionts using a microscope slide. Organisms were sorted by phyla (i.e., bryozoa, hydrozoa, rhodophyta, chlorophyta, and polychaeta [i.e., sessile spirorbid polychaetes]) using a dissecting scope. Once sorted, the epibionts and seagrass blades were placed in separate foil bags and dried in a drying oven at 60°C to constant weight to calculate the epiphyte: *Z*. *marina* biomass relationship.

### Mobile epifauna study

In August 2016, we sampled within three regions of Akkeshi-ko estuary (Fig 1), and within each region we took five samples from a reference seagrass site (no aquaculture) and five samples from a seagrass site with aquaculture (within 1 m of culturing lines). All six sites were between 0.7 and 1.1 m deep. Each pair of sites within each region was separated by 200–500 m, and reference sites were at least 100 m from any aquaculture lines. At each site, we collected three types of samples: 1) seagrass mobile epifaunal samples; 2) seagrass Chlorophyll *a* (chl a) epiphyte samples; and, 3) sediment organic matter (SOM) samples. For the seagrass epifaunal samples, we used 500 μm mesh bags to collect all seagrass shoots inside five randomly placed 50 x 50 cm quadrats at each site. Samples were carefully floated into the mesh bags to reduce loss of any organisms, and samples were stored in buckets on the boat until they could be transferred to the refrigerator at the Akkeshi Marine Station, where they were processed within one week. For the chl a samples, we randomly selected five individual seagrass blades that were transferred to Ziploc bags and stored in a cool dark bucket for transport back to the laboratory, where they were processed within 24 hours. Finally, we took three SOM samples at each site using a plastic corer (5-cm diameter) to 5-cm depth. SOM samples were frozen at -20°C until they could be processed.

The mobile epifaunal samples were processed in the laboratory by washing the contents of the mesh bag into a sorting pan, and then cleaning each seagrass blade of epiphytes and epifauna. We counted the total number of seagrass shoots and took morphological measurements for the first five shoots as above. All cleaned seagrass was placed in foil bags and dried at 60°C to constant weight. The remaining contents of the pan were washed onto a 500 μm sieve, transferred to jars, and preserved in 70% Ethanol until they could be sorted. Epifauna were identified to the lowest taxonomic level and enumerated using a dissection microscope.

We used chl a as a proxy to quantify epiphyte biomass by normalizing chl a concentration to the unit surface area of each shoot collected. Each of our chl a seagrass samples was processed in a dark room, where we used a microscope slide to carefully remove epiphytes from the blades and transfer them to a small amount of filtered seawater. Chlorophyll *a* concentration of the epiphytes was then obtained via the non-acidification method of Welschmeyer [41]. The chl a samples were filtered through Whatmann GF/F glass-fiber filters, and we used a 6 ml N, N-dimethylformamide extraction for 24-hours [42]. The filters were stored in the dark at -20°C until analysis when chl a concentration was determined using a fluorometer (Turner Designs 10-AU-005-CE, Sunnyvale, CA, USA). Cleaned seagrass shoots were individually dried in foil packets at 60°C to constant weight, and we used a biomass to surface area conversion (y = 180.28*x* + 8.309; calculated from 64 local seagrass samples, R^2^ = 0.96) to convert seagrass dry biomass values to SA (cm^2^).

Sediment organic matter samples were thawed to room temperature prior to processing, and percent organic content was assessed using the Loss on Ignition technique [43]. Thawed homogenized samples of approximately 15 g (wet weight) were placed into pre-combusted and weighed crucibles and dried in an oven for 48 h at 60°C to obtain dry weight. The samples were placed in the muffle furnace at 500°C for 5 h to obtain ash-free dry weight.

### Statistical analyses

For the sessile epibiont study, we took an average of each site’s metrics and used a one-way ANOVA with treatment (high-density, low-density, and natural) as a categorical factor to examine differences. Epibiotic community data were analyzed by phylum, and we used the epiphyte biomass to *Z*. *marina* ratio (mg g^-1^) to evaluate differences between communities based on treatment. We visualized community differences using nMDS and followed this with a PERMANOVA of the epibiont matrix (15 samples x 5 phyla), using 4999 permutations and a Bray Curtis distance measure (“vegan” package R)[44]. We also used the Similarity Percentage (SIMPER; “vegan” package R)[44] method to determine the groups that contributed most to among-treatment dissimilarity and these groups were analyzed separately using one-way ANOVA as above. When the effect of treatment was significant, we followed the ANOVA with Tukey’s post-hoc tests (adjusted for multiple comparisons).

For the mobile epifauna study, we used a two-way ANOVA to analyze the seagrass morphology, Chl a, and percent organic content metrics with both region and treatment as fixed effects. While region is a random effect conceptually, because there were only three levels to the variable, treating it as a random effect is thought to be ineffective [45] and including it as a fixed effect allowed us to examine differences between the three regions. For epifaunal community analyses, we excluded rare families that were only present in one sample (n = 4 families). We used nMDS followed by PERMANOVA as above to visualize differences in: 1) the entire epifaunal community (30 samples x 18 families); 2) the entire community minus sessile polychaetes (30 samples x 17 families); and, 3) the amphipod community exclusively (30 samples x 6 families). Amphipods were the only community group with sufficient richness and abundances to conduct a community analysis. To look specifically at differences in faunal abundance between treatments, we used univariate statistics to separately analyze the most dominant faunal groups (i.e., amphipods, isopods, gastropods, and sessile polychaetes). We did not use two-way ANOVA because taxa were not present in all samples; instead, we took an average value for each site and used Welch’s two-sample t-tests.

Across both studies, univariate data were transformed before analysis if they failed to meet the assumptions of normality or homogeneity of variance (Levene’s Test, p > 0.05), with some modest deviations (see Tables 1 & 2 for a list of transformation and deviations). We used a log_(x+1)_ transformation on community matrix data, and Permutational dispersion (i.e., PERMDISP) was used to verify the assumption that multivariate data had equal spread. All analyses were performed in R Studio version 1.1.423 [46].

**Table 1:** One-way ANOVA comparisons and Tukey’s post-hoc tests for the sessile epibiont study.

	df	F-statistic	P-value	Natural mean ± SE (n)	Low-density mean ± SE (n)	High-density mean ± SE (n)
**Canopy height (cm)**	2:12	0.475	0.633	233.0 ± 15.5 (5)	224.4 ± 10.4 (5)	213.3 ± 16.5 (5)
**Blade width (mm)**	2:12	0.674	0.528	8.9 ± 0.6 (5)	9.2 ± 0.6 (5)	8.3 ± 0.3 (5)
**Stem density (shoots m**^**-2**^**)**^a^	2:12	0.664	0.533	42.5 ± 16.0 (5)	36.8 ± 8.4 (5)	51.0 ± 10.0 (5)
**Above-ground biomass (g dry wt m**^**-2**^**)**	2:12	2.258	0.147	93.6 ± 9.4 (5)	79.9 ± 8.2 (5)	68.8 ± 7.1 (5)
**Red algae biomass (mg/g)**^b^	2:12	1.815	0.205	21.1 ± 11.8 (5)	4.7 ± 3.0 (5)	1.5 ± 1.1 (5)
**Sessile polychaete biomass (mg/g)**^a^^,^^c^	2:12	4.863	0.028	0.6 ± 0.5 (5)	8.2 ± 6.3 (5)	7.7 ± 4.8 (5)
Low-High			0.886			
Natural-High			0.075			
Natural-Low			0.032			

^a^Log_10_ transformation

^b^Log_(x+1)_ transformation

^c^Slightly non-normal (Shapiro Wilkes, p = 0.02)

**Table 2:** Two-way ANOVA comparisons and Tukey’s post-hoc tests for the mobile epifauna study.

	DF	Mean sq.	F-statistic	P-value
**Canopy height (cm)**				
Treatment	1	2635	2.412	0.133
Region	2	31274	28.63	**< 0.001**
Treatment*Region	2	1039	0.951	0.401
Residuals	24	1092		
Region1-Region2				**< 0.001**
Region1-Region3				**< 0.001**
Region2-Region3				0.924
**Blade width (mm)**				
Treatment	1	0.511	0.555	0.463
Region	2	8.776	9.529	**< 0.001**
Treatment*Region	2	1.481	1.608	0.221
Residuals	24	0.921		
Region1-Region2				**0.001**
Region1-Region3				**0.005**
Region2-Region3				0.851
**Stem density (shoots m**^**-2**^**)**^a^				
Treatment	1	13.33	0.342	0.564
Region	2	86.93	2.233	0.129
Treatment*Region	2	6.93	0.178	0.838
Residuals	24	38.93		
**Above-ground biomass (g dry wt m**^**-2**^**)**^b^				
Treatment	1	0.010	0.194	0.664
Region	2	1.079	22.12	**< 0.001**
Treatment*Region	2	0.020	0.404	0.672
Residuals	24	0.049		
Region1-Region2				**< 0.001**
Region1-Region3				**< 0.001**
Region2-Region3				0.754
**Epiphyte biomass (ug Chl-A cm**^**-2**^**)**^b^				
Treatment	1	0.607	13.979	**0.001**
Region	2	0.143	3.302	0.054
Treatment*Region	2	0.071	1.638	0.215
Residuals	24	0.043		
**SOM (%)**				
Treatment	1	3.524	6.245	**0.028**
Region	2	0.057	0.100	0.905
Treatment*Region	2	3.006	5.327	**0.022**
Residuals	12	0.564		

^a^Slightly non-normal (Shapiro Wilkes, p = 0.01)

^b^Log_10_ transformation

## Results

### Sessile epibiont study

**Fig 3:**
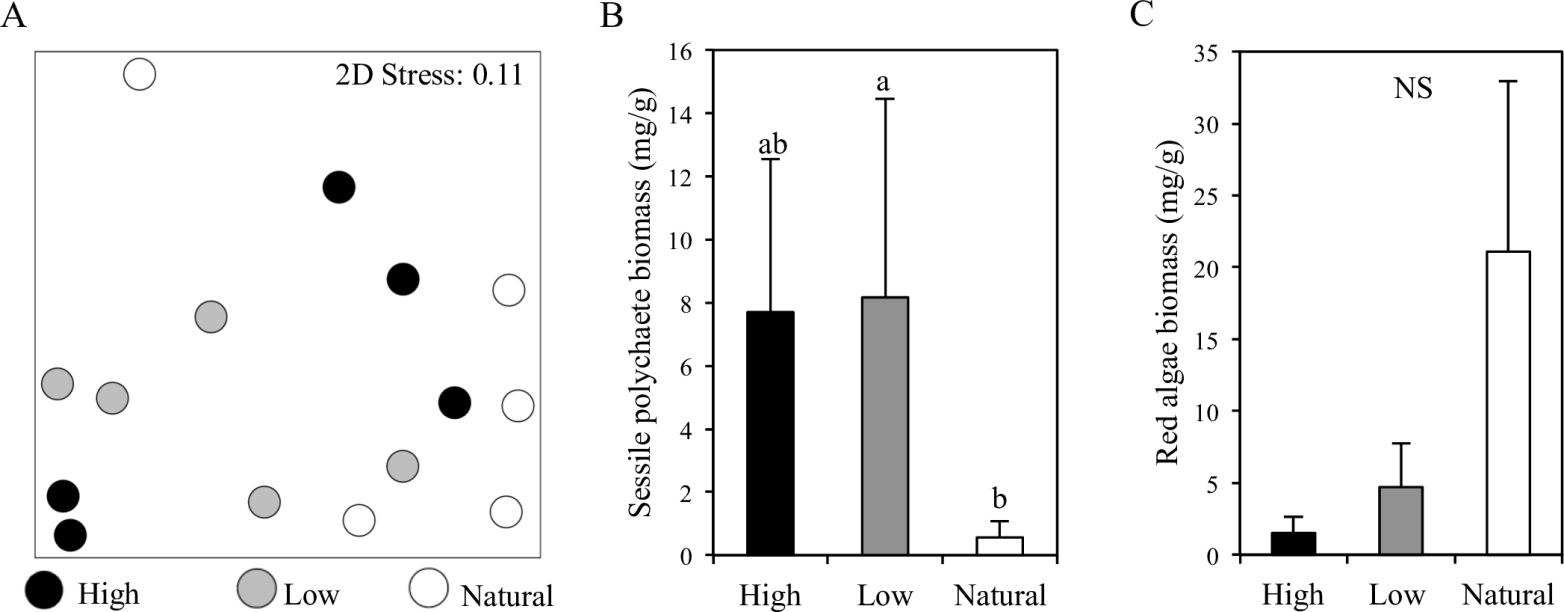
Seagrass beds beneath aquaculture have higher sessile polychaete biomass than reference seagrass beds. NMDS plot showing a Bray-Curtis similarity matrix of biomass for eelgrass-associated fouling phyla (A). Mean (± SE) biomass of sessile polychaetes (B) and red algae (C) among treatments in the sessile epibiont study. Different lower-case letters denote statistical differences between treatments by Tukey’s post-hoc tests (adjusted for multiple comparisons), whereas NS indicates non-significant results.

In the sessile epibiont study, there were no significant differences among treatments in seagrass canopy height (One-way ANOVA, p = 0.63), blade width (p = 0.53), stem density (p = 0.53), or above-ground biomass (p = 0.15; Table 1). There was weak statistical support for a difference in the composition of the fouling community among the three treatments (PERMANOVA, F_2,12_ = 1.79, p = 0.11), but there was a significant difference in the community composition between natural and aquaculture (combining both low- and high-density aquaculture; F_1,13_ = 3.11, p = 0.03; Fig 3A). SIMPER analyses revealed that red algae and sessile polychaetes were driving these community differences with a cumulative species contribution of 87%, so we analyzed these phyla separately. There was a significant difference in sessile polychaete biomass among treatments (One-way ANOVA, p = 0.03), and post-hoc tests revealed a significant difference between the low-density aquaculture and natural treatments (Tukey’s post-hoc test, p = 0.03), a marginally significant difference between high-density aquaculture and natural (p = 0.08), and no difference between low- and high- density aquaculture (p = 0.89; Table 1; Fig 3B). The mean biomass of sessile polychaetes was thirteen times higher at high- and low-density aquaculture sites than natural sites, though variability was high. In almost the exact opposite trend, the mean value for red algal biomass in natural beds was five times higher than low-density beds and fourteen times higher than high-density beds; however, variance across natural sites was also high, and the statistical support for this difference was weak (One-way ANOVA, p = 0.21; Table 1; Fig 3C).

### Mobile epifauna study

**Fig 4:**
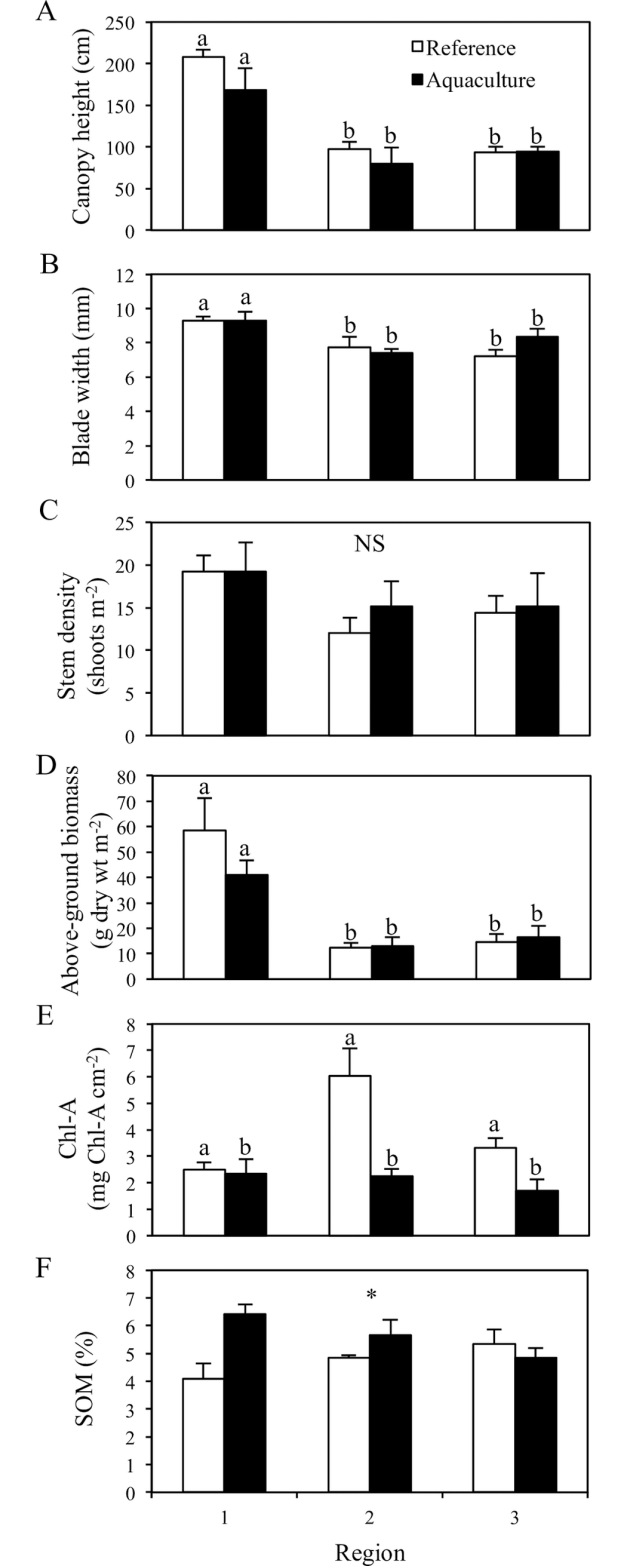
Reference seagrass beds have significantly higher epiphyte biomass than seagrass beds beneath aquaculture. Mean (± SE) seagrass canopy height (A), blade width (B), stem density (C), above-ground biomass (D), epiphyte biomass per cm^2^ (E), and SOM (F) between reference and aquaculture sites. Different lower-case letters denote statistical differences between treatments/regions by Tukey’s post-hoc tests (adjusted for multiple comparisons), NS indicates that there were no differences, and an asterisk indicates a significant interaction.

Between reference and aquaculture areas in the mobile epifauna study, there were no significant differences in seagrass canopy height (two-way ANOVA, p = 0.13), blade width (p = 0.46), stem density (p = 0.56), or above-ground biomass (p = 0.66; Table 2; Fig 4A–4D). There was a significant region effect, however, with the seagrass in region 1 showing higher seagrass canopy heights (two-way ANOVA, p < 0.001), blade widths (p < 0.001), and above-ground biomass (p < 0.001) when compared to regions 2 and 3 (Table 2; Fig 4A–4D). There was a significant difference in epiphyte biomass per cm^2^ of seagrass between treatments (p = 0.001; Table 2). Seagrass in reference areas had a mean epiphyte biomass approximately twice that of seagrass in aquaculture areas (Fig 4E). SOM was significantly different between treatments (p = 0.03); however, this difference was not consistent across regions as there was a significant treatment by region interaction (p = 0.02; Table 2; Fig 4F).

**Fig 5:**
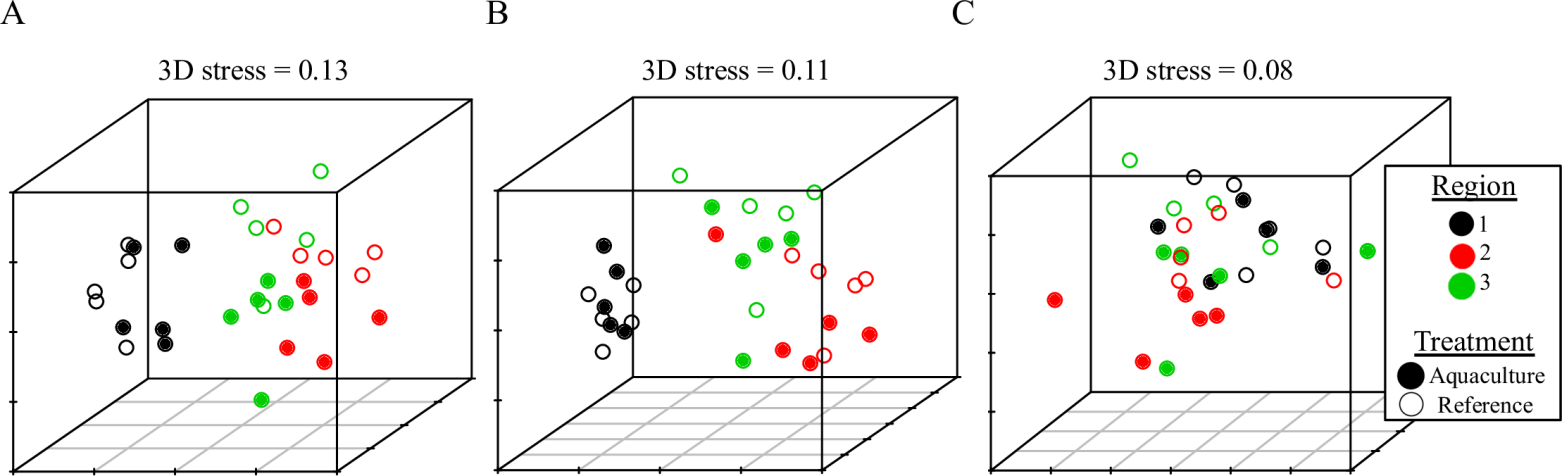
Seagrass epifaunal communities beneath aquaculture are significantly different than reference seagrass communities. NMDS plots showing Bray-Curtis similarity matrices of seagrass epifaunal communities including sessile polychaetes (A), without sessile polychaetes (B), and with only amphipods (C). Open circles are reference sites and filled circles are aquaculture sites.

Sessile spirorbid polychaetes comprised nearly 80% of enumerated organisms across all samples (Table 3), so we visualized and compared community differences with and without their inclusion (Fig 5A and 5B). Including sessile polychaetes, there was a highly significant difference in seagrass epifaunal communities between reference and aquaculture areas (PERMANOVA, F_1,24_ = 4.23, p = 0.004) and also between regions (F_2,24_ = 14.60, p < 0.001) with no interaction (F_2,24_ = 1.68, p = 0.10; Fig 5A). Without sessile polychaetes there were still significant treatment (PERMANOVA, F_1,24_ = 3.09, p = 0.02) and region effects (F_2,24_ = 11.89, p < 0.001) with no interaction (F_2,24_ = 0.74, p = 0.67; Fig 5B). There were significant differences in the amphipod community by treatment (F_1,24_ = 3.93, p = 0.008) and region (F_2,24_ = 6.23, p < 0.001), with no interaction (F_2,24_ = 0.83, p = 0.55; Fig 5C). Amphipods were nearly twice as abundant in eelgrass samples taken from reference areas outside aquaculture than from areas within (Welch’s t-test, t (3) = 4.2, p = 0.02), whereas isopods were nearly twice as abundant in aquaculture areas (t (4) = -6.4, p = 0.003; Table 3). There were no differences in the abundances of sessile polychaetes (t (4) = -1.2, p = 0.29) or gastropods (t (4) = 0.01, p = 0.99; Table 3).

**Table 3:** Total number of epifauna collected in each region in the mobile epifauna study. R = reference sites and A = aquaculture sites.

	Region 1		Region 2		Region 3	
Site type	R	A	R	A	R	A
**Bivalves**	**2**			**1**		
*Macoma calcarea*	*2*					
*Neomysis* sp.				*1*		
**Amphipods**	**76**	**30**	**69**	**55**	**83**	**38**
*Ampithoe lacertosa*	*1*		*6*	*2*	*1*	*4*
*Ampithoe* spp.	*3*	*1*	*4*	*5*	*10*	*3*
*Ampithoe valida*	*7*	*3*	*36*	*20*	*3*	
*Aoroides curvipes*	*8*	*1*	*11*	*1*	*13*	*7*
*Caprella* spp.	*47*	*12*			*1*	*2*
*Corophiidae* spp.	*1*	*1*	*7*	*20*	*1*	*9*
*Grandidiella japonica*		*1*				
*Lysianassidae* spp.	*1*	*1*				
*Pleustes* sp.		*1*				
*Pontogeneia rostrata*	*8*	*9*	*5*	*7*	*54*	*13*
**Gastropods**	**2059**	**859**	**19**	**107**	**5**	**2**
*Alvania concinna*	*2*	*1*			*1*	
*Ansola angustata*			*18*	*99*		
*Lacuna decorata*	*2037*	*858*		*1*		*1*
*Lacuna turrita*	*19*		*1*	*1*	*4*	
*Nassa hypolia*				*1*		
*Siphonacmea oblongata*	*1*			*5*		*1*
**Isopods**	**2**	**5**	**1**	**5**	**2**	**4**
*Cymodoce japonica*	*1*	*1*	*1*	*2*	*1*	*3*
*Idotea ochotensis*	*1*	*1*				
*Paranthura* sp.		*3*		*3*	*1*	*1*
**Platyhelminthes**			**8**		**4**	**5**
*Rhabdocoela* sp.			*8*		*4*	*5*
**Polychaetes**	**8**	**7**	**2**		**2**	**6**
*Harmothoe* cf. *extenuata*			*1*			
*Platynereis bicanaliculata*	*7*	*2*	*1*			
*Syllidae* sp.	*1*	*5*			*2*	*6*
**Sessile Polychaetes**	**90**	**302**	**59**	**6199**	**1358**	**534**
*Neodexiospira brasiliensis*	*90*	*302*	*59*	*6199*	*1358*	*534*
**Total**	**2240**	**1221**	**158**	**6375**	**1460**	**596**

## Discussion

Our study revealed that long-line oyster aquaculture had no detectable effects on seagrass morphology, but did result in reduced algal epiphyte biomass and a difference in epifaunal community abundance and biomass in Akkeshi-ko estuary, Japan. Some of the most commonly referenced adverse effects of bivalve aquaculture on seagrass beds are bottom shading [29], physical disturbance caused by harvesters or the aquaculture gear itself [28], and competition for space [25, 47]; these stressors generally vary with environmental context and gear, but most are likely to be reduced (perhaps to elimination) with long-line culture as practiced in Akkeshi-ko. Some alternate forms of oyster aquaculture have been shown to result in reduced *Z*. *marina* biomass and density [48, 49], which has been attributed to seagrass light limitation. The only structures on the surface of the water in Akkeshi-ko are small buoys and lines, and in most areas of the estuary the seagrass canopy reaches the water surface, so it is unlikely that the oysters in our system are causing substantive shading to the seagrass. Furthermore, the oysters in Akkeshi-ko never come into direct contact with the seabed and they are harvested by hand, thus reducing physical bottom disturbance and competition for space.

Since long-line oyster aquaculture in Akkeshi-ko avoids many of the direct mechanisms of concern to seagrass found with alternate forms of culture, it is perhaps not surprising that we found few direct effects in this study. Nevertheless, both plant and animal epibionts can exert strong control on the productivity of their host macrophyte [50], and therefore any environmental changes that have a direct effect on epibiont biomass or community composition may have indirect effects on the seagrass itself. Considering this, it is possible that the aforementioned stressors (e.g., shading, physical disturbance, competition for space) introduced by long-line aquaculture *are* negatively affecting the seagrass in our system, but that those effects are being ameliorated via facilitative interactions between the oysters and seagrass. Both of our studies yielded evidence that reference seagrass beds outside aquaculture had higher epiphyte loads. While there was high variation between sites in the sessile epibiont study and the results were not statistically significant, reference areas had an average red algal epiphyte biomass 14 times higher than high-density aquaculture and five times higher than low-density aquaculture beds. Similarly, we found higher epiphyte biomass per cm^2^ in our mobile epifauna study. Peterson and Heck [22] predicted that epiphyte biomass in *Thalassia testudinum* beds would be lower in the presence of filter-feeding mussels because of elevated populations of epiphyte grazers utilizing the increased structure of the mussels. While they did find lower epiphyte biomass in conjunction with mussels, they did not find increased grazer populations and thus they concluded that the mussels themselves might have been feeding on the epiphyte propagules before they could settle on the seagrass. We found a similar trend in our data, with lower epiphyte biomass and lower populations of amphipods in seagrass beds under aquaculture, suggesting that amphipods were not solely responsible for the lower epiphyte biomass observed in aquaculture areas. Isopods are also well-known epiphyte grazers [51], and we found evidence suggesting that isopod populations were elevated in aquaculture areas (though we counted less than 20 isopods across all sites), so it is possible they were responsible for some epiphyte consumption.

In two studies on the gut contents of cultured oysters in Akkeshi-ko estuary, Kasim & Mukai [52, 53] found that benthic diatoms (including those found on eelgrass blades) made up nearly 70% of oyster diets. These researchers also found that the composition of diatoms in the water column did not match the composition in gut contents, showing that oysters are capable of preferentially feeding on certain species of diatoms, even when they are relatively rare. While farmed oysters appear to feed on epiphytic microalgae, it is unclear whether this also applies to epiphytic macroalgal propagules. Nevertheless, cultured oysters may at least in part be responsible for the reduced epiphyte loads in aquaculture areas, but more work is needed to verify this mechanism.

A reduction in epiphyte biomass could have positive effects on the seagrass itself and its ability to photosynthesize, but could have unknown community-wide effects. For example, we found that sessile spirorbid polychaete biomass was significantly higher in aquaculture beds versus natural beds in the sessile epibiont study. Rönnberg *et al*. [54] similarly found an increased number of sessile organisms growing on *Fucus vesiculosus* fronds with decreasing algal epiphytes, and other studies have shown that certain species of sessile polychaetes preferentially settle away from macroalgal epiphytes where competition for space is less intense [55, 56]. On one hand, algal epiphytes are generally regarded as a hindrance to their macrophyte hosts because they can reduce incoming light by up to 80% [54]. On the other hand, algal epiphytes are an important link in the food chain [57] and are largely responsible for transferring energy between trophic levels [58]. Furthermore, epiphytes increase structural complexity and provide refuge for many organisms, and thus any change in epiphytes could have a strong effect on mobile epifaunal community structure. In fact, Momota & Nakaoka [59] found that sessile epibiont biomass (particularly red branching algae and spirorbid polychaetes) were a stronger predictor of the mobile epifaunal community composition than abiotic factors in Akkeshi-ko estuary. A positive or negative value judgment of a change in epibiont community composition is beyond the scope of this paper, but it is likely that the red branching epiphytes most commonly observed in our study cause more shading to the seagrass itself than do sessile polychaetes. If this is true, a reduction in epiphyte biomass (even accompanied by an increase in sessile polychaete biomass) could reduce seagrass light limitation and facilitate growth. Either way, our data offer evidence that the effects of aquaculture are complex and expressed at the community level.

A notable limitation of our study is a lack of before data. Unfortunately, given the long history of aquaculture in Akkeshi-ko, quantitative data on seagrass and associate flora and fauna before the advent of aquaculture do not exist. Long-line aquaculture in Akkeshi-ko increased more than 10-fold between 1990 and 2004 (but production has remained stable since then [37]). Despite this expansion, areal seagrass extent (quantified via historic remote sensing) has also increased [60], suggesting no direct negative impacts of aquaculture on overall seagrass extent. In fact, it is possible that the seagrass has expanded due to increased water clarity from the higher densities of oysters [23]. Oysters in Akkeshi-ko are grown at moderate densities, employing traditional methods and small boats, and utilizing minimally invasive harvesting techniques. Additionally, both *C*. *gigas* and *Z*. *marina* are native to Akkeshi-ko, and therefore it is possible that they are particularly well adapted to their environment and to one another [61]. Finally, we did detect significant differences in seagrass size and epifaunal community among regions. This is consistent with other studies in Akkeshi-ko that found seagrass near region 1 to be significantly larger than in other areas of the estuary [59]. The mechanism for this is not entirely clear, but probably relates to the proximity of region 1 to the inlet. Future research should investigate any potential interactions between aquaculture impacts and abiotic factors.

There are many potential costs and benefits to bivalve aquaculture that must be considered in their specific contexts and locations, and more work is needed to elucidate whether the community-level effects documented in Akkeshi-ko estuary hold across different seasons and environmental settings. Sustainable aquaculture production that can meet growing demands, but that also has minimal impacts on coastal habitats will be predicated on responsible management of practices, as well as proper selection of culturing locations and techniques that minimize conflicts and habitat trade-offs and maximize facilitations. Long-line aquaculture may be a preferable technique when trying to minimize impacts to seagrass beds because it avoids many of the major concerns put forth like bottom shading, physical disturbance, and competition for space.
